# Fronto-parietal cortex activation during walking in patients with Parkinson's disease adopting different postural strategies

**DOI:** 10.3389/fneur.2022.998243

**Published:** 2022-10-24

**Authors:** Xinyuan Zhang, Yue Wang, Jiewei Lu, Jin Wang, Zhilin Shu, Yuanyuan Cheng, Zhizhong Zhu, PeiPei Liu, Yang Yu, Ningbo Yu, Jianda Han, Jialing Wu

**Affiliations:** ^1^Clinical College of Neurology, Neurosurgery and Neurorehabilitation, Tianjin Medical University, Tianjin, China; ^2^College of Artificial Intelligence, Nankai University, Tianjin, China; ^3^Tianjin Key Laboratory of Intelligent Robotics, Nankai University, Tianjin, China; ^4^Department of Neurorehabilitation and Neurology, Tianjin Huanhu Hospital, Tianjin, China; ^5^Department of Rehabilitation Medicine, Tianjin Huanhu Hospital, Tianjin, China; ^6^Tianjin Key Laboratory of Cerebral Vascular and Neurodegenerative Diseases, Tianjin Neurosurgical Institute, Tianjin, China

**Keywords:** Parkinson's disease, functional near-infrared spectroscopy, dual task, postural strategy, Gait

## Abstract

**Background:**

Cortical activation patterns in patients with Parkinson's disease (PD) may be influenced by postural strategies, but the underlying neural mechanisms remain unclear. Our aim is to examine the role of the fronto-parietal lobes in patients with PD adopting different postural strategies and the effect of dual task (DT) on fronto-parietal activation.

**Methods:**

Two groups of patients with PD adopting either the posture first strategy (PD-PF) or the posture second strategy (PD-PS) were examined respectively when in the “OFF” state while single-walking task (SW) and DT. Frontal and parietal lobe activity was assessed by functional near infrared spectroscopy (fNIRS) and measuring gait parameters. Linear mixed models were used for analyses.

**Results:**

Patients with PD who adopted PS had greater cortical activation than those who adopted PF, and there was no difference between PF and PS in the behavioral parameters. For oxyhemoglobin levels, the task condition (SW vs. DT) had a main effect in fronto-parietal lobes. Postural strategy (PD-PF vs. PD-PS) a main effect in the left prefrontal cortex (LPFC), left parietal lobe (LPL), and right parietal lobe (RPL) regions. In the task of walking with and without the cognitive task, patients with PD adopting PS had higher activation in the LPL than those adopting PF. In DT, only PD patients who adopted PS had elevated oxyhemoglobin levels in the LPFC, right prefrontal cortex (RPFC), and LPL compared with the SW, whereas patients with PD who adopted PF showed no differences in any region.

**Conclusion:**

Different patterns of fronto-parietal activation exist between PD-PF and PD-PS. This may be because PD-PS require greater cortical functional compensation than those adopting PF.

## Introduction

Patients with Parkinson's disease (PD) have impaired dual-task (DT) postural control as a result of reduced processing resources and deficits in basal ganglia mechanisms (1, 2), causing disability and increased fall risk, which can lead to loss of independence (3). In challenging cognitive-motor DT conditions, healthy people can correctly sequence the two tasks by motivation to reduce danger and increase pleasure (4), whereas patients with PD are more dependent on goal-directed behaviors due to reduced automaticity, resulting in an inability to correctly judge the risk of their behaviors and thus achieve a reasonable sequence (5). Patients with PD seem to adopt a posture second strategy (i.e., prioritizing secondary cognitive tasks over postural control when performing dual tasks) during complex DTs, treating the elements of the tasks with the same priority (3) or prioritizing the second task (6), which can impair balance and lead to falls. It is widely accepted that patients with PD should adopt a posture first strategy (i.e., prioritizing postural control over secondary cognitive tasks when performing dual tasks) to compensate for balance deficits and to reduce the risk of falls (7, 8). However, it has also been shown that concentrating on posture while performing dual tasks does not necessarily improve postural stability and may even lead to degradation of motor automaticity or flexibility (9, 10). Studying the prioritization process of patients with PD during DT may provide vital information to help adapt interventions.

It has been proposed that patients with PD have limited attentional resources, which leads to their reduced ability to perform multiple tasks simultaneously (11). Another explanation is that their attentional resources are relatively intact, but they perform the task with less automation than healthy individuals (12). Therefore, increasing prefrontal activation through attentional processes and executive resources is needed by patients with PD to compensate for gait automaticity (13). Existing studies suggest that in accordance with the task prioritization model, the postural strategy of patients with PD is based on the patient's postural reserve (an individual's ability to respond most effectively to postural threat) (14). Postural reserve is influenced mainly by postural control and automaticity (15), whereas postural control is primarily controlled by the sensory feedback system in the parietal lobe and is further enabled by other higher-level cortical control functions, such as sensorimotor integration, adaptation, and anticipatory mechanisms (16). Impaired motor automaticity is a prominent feature of patients with PD; basal ganglia dysfunction leads to reduced motor automaticity, resulting in greater reliance on attention and executive resources to control movement (17, 18). In combination, these results suggest that the pattern of cortical activation in patients with PD may differ across postural strategies (11, 13, 19); however, the neural mechanism is unclear. Past studies have shown that gait and cognitive activity share a fronto-parietal network; both frontal and parietal lobes are associated with control of gait and play an important role in managing the concurrent execution of tasks (20, 21). Hence, assessing fronto-parietal activation is critical to understanding the involvement of cortical circuits this mechanism.

We hypothesized that different postural strategies in patients with PD have different cortical activation patterns during the single-walking task (SW) and DT, and patients who adopt the posture second strategy (PD-PS) have greater cortical activation than patients who adopt the posture first strategy (PD-PF). To examine this hypothesis and better understand the impact of postural strategies on fronto-parietal activation during walking, we analyzed patients with PD using functional near-infrared spectroscopy (fNIRS) to assess fronto-parietal cortex activation during walking. Consequently, the aims of this study were to (a) examine the differentiation of fronto-parietal activation in patients with PD adopting different postural strategies during DT and (b) investigate the effect of DT on fronto-parietal activation in patients with PD adopting different postural strategies.

## Methods

### Participants

Forty patients with PD were referred for inclusion in the study. The inclusion criteria for patients with PD were that they: (a) fulfilled the Movement Disorder Society (MDS) clinical diagnostic criteria for PD in 2015 (22); (b) were compatible with stages I to III of the Hoehn and Yahr Scale (H&Y); (c) were able to walk at least 5 min independently; and (d) had taken stable medications for the past month. Exclusion criteria included (a) history of neurological or psychiatric disorders other than PD; (b) diagnosis of cognitive impairment or dementia [based on a Mini-Mental State Examination (MMSE) score <24]; and (c) presence of any musculoskeletal or cardiac and pulmonary conditions, or painful conditions, which may affect gait or balance. In addition, each patient was checked for blood pressure in the prone and standing positions, and patients with postural hypotension were excluded. All participants provided written informed consent before participating in the research. This study was approved by the Tianjin Huanhu Hospital ethics committee and was registered in the Chinese Clinical Trial Registry.

The patient groups consisted of 12 PD-PF and 28 PD-PS, assessed using the modified Attention Allocation Index (mAAI) which measures the ability to allocate attention in response to instructed focus in the “OFF” state (dopaminergic drugs withdrawn for 12 h or more) to assess trade-offs within each task (23, 24), with positive mAAI values indicating the posture second strategy and negative values indicating the posture first strategy (24). Both groups were assessed for demographic characteristics and cognitive status. The Movement Disorders Society United Parkinson's Disease Rating Scale part III (MDS-UPDRS III) was applied to measure motor symptom severity in the “OFF” state. Cognitive function assessment was evaluated using MMSE when participants were in the “ON” state.

### Procedures

Fronto-parietal cortex activation and behavioral tests were both assessed during the “OFF” medication state. Behavioral tests and fronto-parietal activity were assessed under three conditions: SW, i.e., walking back and forth along a 10 m pathway at a comfortable speed; single cognitive task (SC), performing sequential subtraction of seven from a random three-digit number given prior to the trial; and DT, which implies walking while subtracting. Each task was performed three times in a pseudo-randomized order. Each trial began with 30 s of quiet standing time with instructions not to speak or move the head. After these 30 s, the instruction “start” (for SW), “(a given number)” (for SC), or “start with (a given number)” (for DT) was given. Next, the participants performed the task for 35 s, and then they were instructed to stop and stand quietly for another 20 s, after which the participants were provided breathing space according to their needs. Before each trial, the participant was required to stand still for at least 1 min to minimize the fluctuations in blood pressure. The participants were instructed to complete the motor task in the most natural way and the cognitive task in the fastest and most accurate manner. These instructions were designed to encourage participants to give equal priority to motor tasks and cognitive tasks.

### Behavioral task performance

Participants wore six inertial sensors (Opals by APDM Wearable Technology—an ERT company, Portland, Oregon, United States) using nylon buckle straps on the following body parts: one on the back of each foot, one on each wrist, one on the sternum, and one on the lower back. The data output was synchronized among all Opal sensors and consisted of a three-axis accelerometer, gyroscope, and magnetometer that were combined to calculate the spatial orientation. Data were collected at 128 Hz, and gait parameters were extracted. To show the gait performance of SW and DT, stride length was analyzed and presented as a key metric for walking. The performance on the subtraction task was assessed by counting the number of correct responses for each trial. Finally, the cognitive dual-task effect (cDTE) and motor dual-task effect (mDTE) were calculated for each participant in the three conditions. The mDTE variable was calculated by the step length in SW and DT and cDTE by the number of correct responses in SC and DT. The magnitude of the dual-task effect (DTE) can be expressed according to the formula (23):


$$\begin{array}{l} DTE=\frac{\text{single task }-\text{ dual task}}{\text{single task}}\times 100\% \end{array}$$

Task prioritization was measured using the mAAI, with a positive value indicating posture second strategy (i.e., mDTE is greater than cDTE and patients focus more attention on cognitive tasks when dual-tasking) and a negative value indicating posture first strategy (i.e., cDTE is greater than mDTE and patients focus more attention on gait tasks during DT). The mAAI was calculated using the following equation:


$$\begin{array}{l} \text{mAAI}=\text{mDTE}-\text{cDTE} \end{array}$$

### Functional near-infrared data recording

**Figure 1:**
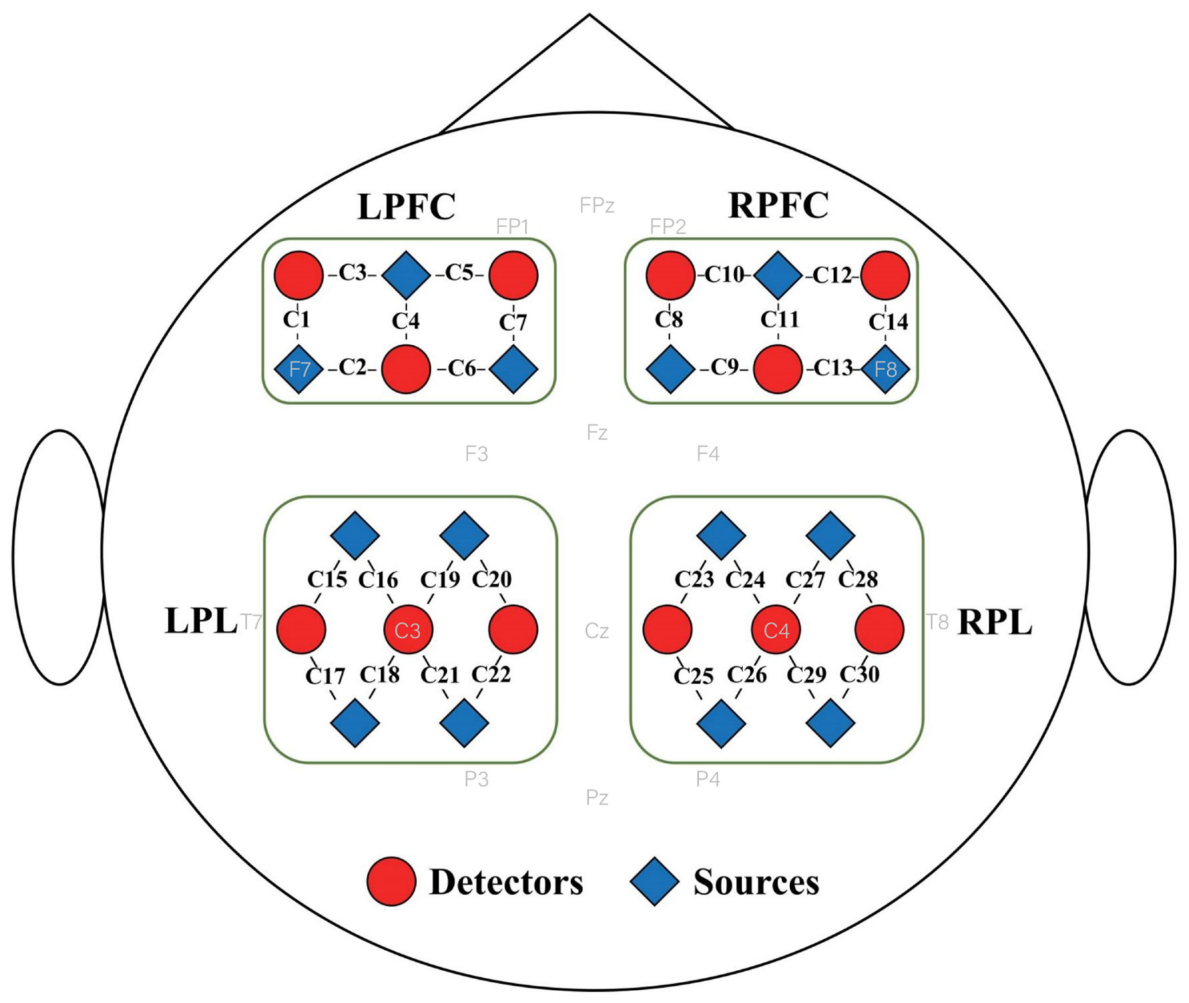
Distribution of fNIRS optodes. Twenty-six fNIRS optodes, including 14 sources and 12 detectors, were placed on four brain regions, namely the left and right prefrontal cortices (LPFC and RPFC) and left and right parietal lobes (LPL and RPL), resulting in 30 channels. The sources and detectors are represented by red triangles and blue circles; respectively.

A portable fNIRS system (NirSmart, Danyang Huichuang Medical Equipment Co., Ltd., China) with wavelengths of 730 nm and 850 nm was used to record the oxygenated hemoglobin concentration change (ΔHbO_2_) with a sampling rate of 11 Hz. The fNIRS system consists of 26 optodes, including 14 sources and 12 detectors. The fNIRS optodes were placed on the left and right prefrontal cortices (LPFC and RPFC) and parietal lobes (LPL and RPL) according to the 10–20 electroencephalogram (EEG) system, resulting in 30 channels. The optode distribution is shown in Figure 1. The source-detector distance was 3 cm. The participants wore a black hood to prevent the interference of surrounding light on the optical signals.

The fNIRS data were preprocessed using NirSpark software package (Danyang Huichuang Medical Equipment Co., Ltd China) and Matlab R2021a. The recorded brain signals were converted into ΔHbO_2_ signals in accordance with the modified Beer-Lambert law (25). The converted signals were filtered using a 0.01–0.2 Hz bandpass filter to remove physiological noise (26). To remove motion artifacts, the filtered signals were processed using an approach based on the moving standard deviation and cubic spline interpolation. The signal quality was checked by visual inspection. The processed signals during walking were extracted and referred to a 5 s baseline before walking. Since the hemodynamic response of fNIRS is temporally delayed (~3 s) from the onset of the underlying neural activity (27, 28), the processed ΔHbO_2_ signals between 3 and 33 s during walking were extracted. All fNIRS channels were included in the data analysis, the signal was analyzed by the mean of each channel in each cerebral hemisphere and each trial. Each baseline concentration was subtracted from the average concentration during task performance to assess the relative change in HbO_2_ concentration under each experimental condition.

### Statistical analysis

Statistical analyses were performed using SPSS version 21, using an α level of *p* ≤ 0.05 to determine statistical significance. Dependent variables were described as means and standard deviations. Differences in oxygenated hemoglobin (HbO_2_) levels between groups (i.e., PD-PF and PD-PS) and task conditions (i.e., SW and DT) were analyzed by applying linear mixed models, including interactions between groups and tasks. The models explained the within-subject nature of the task through random effects on participants. Posture strategy and task state were used as fixed factors with unstructured repeated covariance types and maximum likelihood estimates. The models for HbO_2_ levels also included age and MDS-UPDRS III as covariates to control for the influence of variance on motor progression in patients with PD. Linear mixed models of HbO_2_ concentrations included a random intercept for each participant, as this improved the fit of the model. For all models, the variance matrix was set to the variance components.

## Results

### Participant characteristics

The participants' characteristics are shown in Table 1. No differences in age, sex, education, disease duration, or H&Y stage were observed between groups. There was no difference in scores on the MDS-UPDRS III and MMSE between the two groups.

**Table 1:** Participant characteristics.

	**Posture first** **(*n* = 12)**	**Posture second** **(*n* = 28)**	***P*-value**
Age, years, mean (SD)	62.00 (9.17)	65.89 (6.90)	0.147
Disease duration, years, mean (SD)	4.08 (2.86)	4.96 (3.33)	0.43
Sex, male/female	8/4	13/15	0.311
Height, cm, mean (SD)	166.17 (6.28)	167.11 (7.85)	0.716
Weight, kg, mean (SD)	72.75 (8.58)	68.32 (9.26)	0.615
Education, years, mean (SD)	10.42 (4.74)	12.00 (3.27)	0.229
MMSE, 0–30, mean (SD)	27.42 (1.98)	27.61 (2.02)	0.785
MDS-UPDRS III “OFF”, 0–132, mean (SD)	37.92 (12.28)	33.79 (11.55)	0.315
H&Y stage (I/II/III)	(1/3/8)	(1/7/20)	0.713
Levodopa equivalent dose, mg/d, mean (SD)	445.58 (228.99)	494.75 (206.66)	0.508

H&Y, Hoehn and Yahr scale; MDS-UPDRS III, Movement Disorders Society—Unified Parkinson Disease Rating Scale, motor part; MMSE, Mini Mental State Examination; SD, standard deviation.

### Behavioral results

A summary of gait changes is shown in Table 2. A main effect emerged in the walking condition, reflecting the effect of DT on gait (i.e., SW vs DT) (stride length *p* < 0.001, step speed *p* < 0.001). No interaction between postural strategy and walking condition was observed for the above gait parameters (*p* > 0.20). *Post hoc* analyses revealed a significant deterioration of spatial-temporal metrics during DT in the PD-PF (stride length *p* < 0.001; stride speed *p* < 0.001; subtraction *p* < 0.01) and PD-PS (stride length *p* < 0.001; stride speed *p* < 0.001) groups, but a significant improvement in subtraction performance during DT in the PD-PS group (*p* < 0.001). There were no statistically significant differences between posture strategy groups in step speed (SW, *p* = 0.109; DT, *p* = 0.103) or cognitive task performance (SC, *p* = 0.140; DT, *p* = 0.673). The step length of the PD-PF group tended to be greater than the PD-PS group (SW, *p* = 0.095; DT, *p* = 0.085).

**Table 2:** Behavioral parameters by groups and task.

**Behavioral parameters**		**Posture first** **(*n* = 12)**	**Posture second** **(*n* = 28)**	**Group comparison**	**Task comparison**	**Interaction**
Stride length, cm, mean (SD)	SW	1.11 (0.16)	1.00 (0.21)	0.08	0.001	0.519
	DT	0.98 (0.18)	0.84 (0.25)			
Gait velocity, cm/s, mean (SD)	SW	1.06 (0.16)	0.95 (0.21)	0.097	0.001	0.584
	DT	0.89 (0.19)	0.76 (0.24)			
Subtraction, *n*, mean (SD)	SC	7.39 (4.40)	5.46 (3.37)	0.581	0.188	0.001
	DT	5.83 (3.99)	6.39 (3.31)			
mDTE, 50% (25%,75%)		0.13 (0.10, 0.18)	0.12 (0.06, 0.21)	0.631		
cDTE, 50% (25%, 75%)		0.23 (0.17, 0.32)	−0.14 (0.62, 0.01)	0.001		
mAAI, 50% (25%, 75%)		−0.08 (-0.22,−0.06)	0.24 (0.12, 0.73)			

SW, single walking; DT, dual task; cDTE, Cognitive dual-task effect; mDTE, Motor dual-task effect; mAAI, Modified attention allocation index; SC, single cognitive task; SD, standard deviation.

### Fronto-parietal cortex activity

**Figure 2:**
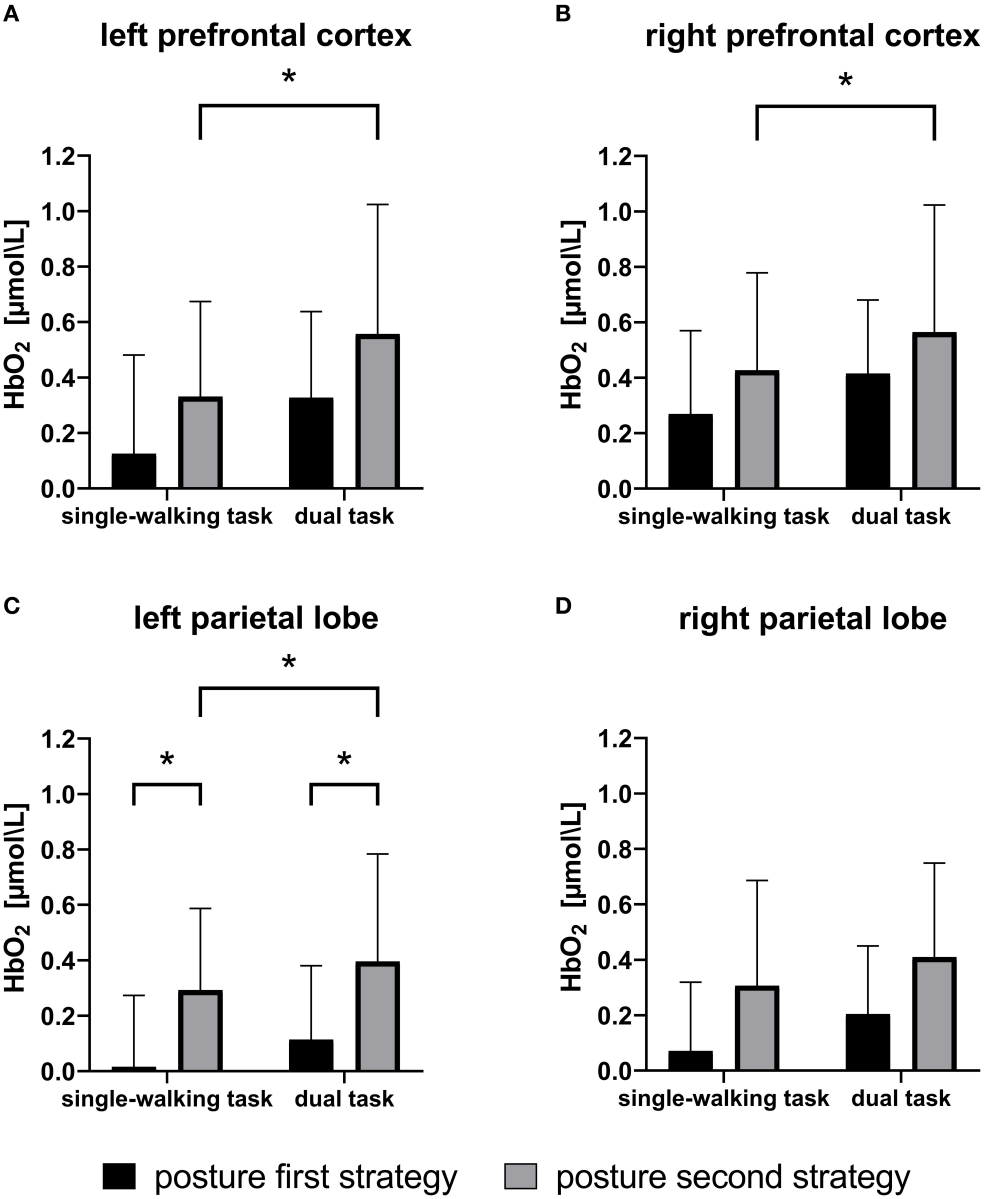
Means and standard deviations of oxyhemoglobin (HbO_2_) levels during single and dual tasks for patients with Parkinson's disease adopting the posture first strategy (PD-PF) and posture second strategy (PD-PS). The PD-PS group had greater cortical activation than the PD-PF group. In single-walking (SW) and dual-tasking (DT) conditions, PD-PS group had higher activation in the LPL than those in the PD-PF group. In DT, only the PD-PS group had elevated HbO_2_ levels in the LPFC, RPFC, and LPL compared with the SW task, whereas participants in the PD-PF group showed no differences in any region. *p < 0.05. LPL, left parietal lobe; LPRC, left prefrontal cortex; RPFC, right prefrontal cortex.

For oxyhemoglobin levels (Figure 2), there was a main effect of walking condition (SW vs. DT) in all four brain regions studied (LPFC, *p* = 0.001; RPFC, *p* = 0.009; LPL, *p* = 0.023; RPL, *p* = 0.028), whereas posture strategy (PD-PF vs. PD-PS) had main effects in the LPFC (*p* = 0.010), LPL (*p* = 0.011), and RPL (*p* = 0.013), and only a trend in the RPFC (*p* = 0.053), with no significant interaction found between posture strategy and task (*p* > 0.2). In the SW (*p* = 0.007) and DT (*p* = 0.028) conditions, the PD-PS group showed higher activation in the LPL than that in the PD-PF group, with the same trend in the RPL (SW, *p* = 0.056; DT, *p* = 0.067). Only the PD-PS group had elevated oxyhemoglobin levels in the LPFC (*p* < 0.001), RPFC (*p* = 0.023), and LPL (*p* = 0.045) regions during the dual task, with a trend in the RPL (*p* = 0.086), while the PD-PF group had no significant differences in any of the four brain regions studied (*p* > 0.2).

## Discussion

To the best of our knowledge, this is the first study to describe the different changes in movement-related oxygenated hemoglobin in the cortical areas of patients with PD adopting different postural strategies. Our results showed that LPL activation was higher in the PD-PS than PD-PF group, suggesting that PD-PS requires a greater contribution from the executive motor network because of impaired motor automaticity. This may indicate that it is not only the subcortical structures that control gait in PD but also in more complex tasks involving conscious attention and probably some ongoing cognitive processing (29, 30). The temporo-parietal association cortex produces cognitive information and forms the basis for maintaining vertical posture and constructing motor programs (31). Cognitive information in the parietal cortex is of importance for accurate gait control, especially when subjects encounter unfamiliar environments (31). Consistent with the idea that gait impairment in PD is due in part to deficits in automaticity, some patients with PD need to shift their behavioral strategies from automatic motor control to goal-directed strategies (5, 32, 33). Patients with PD with posture second strategies may need to compensate for gait deficits through internal cues by using particular self-cueing instructions or by focusing on the intended part of the gait (34, 35), which involves the prefrontal (frontal visual field) and parietal areas (36). Parietal areas may be recruited to save the information of motor sequences and contribute to the timing of sequences, which guarantees that each movement takes place following the successful completion of the previous one and ensures the selection and monitoring of sequences (37, 38). Huang et al. showed that during the execution of dynamic force-matching tasks, the effects of postural strategies were concentrated in CP3 and P3 regions of the LPL (39), which are related to the function of the left parietal cortex in adapting to dissimilar postural conditions and updating the body's spatial awareness. The participants that used PD-PS had higher levels of HbO_2_ in the parietal lobe during SW, possibly reflecting the need to utilize cognitive resources, even in this comparatively simple task.

Our study showed increased fronto-parietal activation in participants when DT was performed, but this was statistically significant only in the PD-PS group. These data indicate that PD-PS requires more prefrontal cortex and parietal cortex activity to compensate for inefficient neural processing during automated motor behaviors (40). PFC activation is considered to play a role in a compensatory mechanism for maintaining task performance (41). Orcioli-Silva et al. compared the success of patients with PD in different studies in terms of cognitive performance when performing DT (11). PFC activation was affected by the strategy applied during DT, with the posture second strategy leading to greater PFC activation. The PFC, middle frontal gyrus, and the parietal lobe (PL) have been demonstrated to correlate with DT (42–45). The PFC, particularly the dorsolateral prefrontal cortex (DLPFC), plays a significant role in allocation and coordination of attentional resources (42). The PL is implicated in attention, working memory, and planning. Several studies have demonstrated that both the premotor cortex and PL participate in motor learning and execution (46–48). To successfully attain precise inter-limb coordination during walking, posture must be optimized before intentional behaviors to maintain balance and stability (31). Consequently, visuo-parieto-frontal cortical projections are crucially engaged in the accomplishment of ongoing purposeful control through anticipatory adjustments of posture (49). Our results suggest that the LPL may be more involved than the RPL; this may be due to functional differences between the left and right hands and functional asymmetries between the dominant and non-dominant hemispheres, with all participants in this study being right-handed (50). A second reason may be that the LPL and RPL play different roles in digit processing, with the LPL being more involved in precise, language-dependent digit processing (51).

We postulate that patients with PD can be effective in improving gait by focusing more attention on walking while performing complex tasks. To lower the risk of falls, it is generally considered that patients with PD should pursue the posture first strategy, which is to say, posture control should be prioritized over other tasks to compensate for balance deficits and prevent falls when DT is inevitable (7, 8). On the contrary, pursuing the posture second strategy, in which the attention is focused on a cognitive task instead of maintaining posture, results in impaired balance performance and postural adjustment (3, 52). Bloem et al. (3) concluded that these patients were incapable of correctly estimating the risk of their actions; therefore, they unwittingly exacerbated the risk of falling in DT situations. According to the task prioritization model, postural reserve is a key factor in postural strategy (14). Hanna et al. showed that postural strategy in PD is related to cognitive levels, and that patients with PD have a mild cognitive impairment (MCI), thus prioritizing cognitive tasks over gait (PD MCI). In contrast, patients with non-MCI focus more on the performance of gait tasks (53). However, our study found no difference in the cognitive levels between these two groups. We also found that the PD-PS group performed better in cognition while DT than in cognition while SW. According to the central capacity-sharing model, when two tasks are performed concurrently, resources must be redistributed (54). Posture second strategy devotes limited resources to cognitive tasks when performing dual tasks, which causes a decline in gait performance. The posture second strategy may be a safe DT strategy in patients with early-stage PD that have no clinically detectable postural change. For these patients, the posture second strategy can be used to lower the negative impact of DT performance and facilitates the automaticity of postural control (10). In contrast, our patients were mainly in stages II–III of the H&Y scale; hence, different conclusions may have been obtained with a different patient group.

Furthermore, given the preliminary evidence that PD-PF and PD-PS fronto-parietal activation patterns differ, this may require different treatment approaches and strategies for gait assessment and training in patients with PD. These findings, that posture first strategies may be optimal, may influence appropriate task prioritization instructions in dual-task gait training and safe walking education in patients with PD. Perhaps focusing more attention on gait tasks during dual-task training may be helpful in helping patients with PD cope with complex daily environments, as it will improve postural stability through enhanced postural automaticity and rely on flexible cognitive resources to perform walking-related tasks in an efficient and safe way (55–57). As a means of preventing falls, it may promote functional independence and improve the standard of living of patients with PD.

There are several limitations worth noting and some suggestions for future research directions. First, the two groups differed significantly in number, with more patients with PD in the posture second strategy group than in the posture first strategy group. This may be a true reflection of the fact that most patients with PD use a posture second strategy during DT, which prioritizes cognitive tasks over maintaining posture. Certainly, evidence for this strategy has also been found in some studies assessing default conditions (in other words, when no clear indication of task priority is given) (58, 59). Additionally, postural strategy and hazard estimations are dynamic in nature; for example, the postural strategy in patients with PD may be notably different in the “ON” and “OFF” states (14). Therefore, it may be necessary to study the cortical activation in patients with PD adopting different postural strategies in the “ON” state of dopaminergic drugs. The cognitive examination of patients with PD during the OFF state is affected by tremor or physical discomfort and can not reflect the true cognitive of patient; therefore, we only perform the MMSE during the ON state, but this may have lost some information. In addition, in the experimental design, the walking task required the patient to turn and continue walking at the 10-meter end, and the cognitive task required that the patient should verbally state the subtraction answer. Turning and verbal expression may affect cortical activation (i.e., produce motion artifacts). In future studies, the fNIRS analysis should be modified to account for these effects.

In conclusion, the results of this study indicate that PD patients who adopt postural second strategy, whether SW or DT, require greater frontal and parietal lobe activation and recruit more cognitive-motor regions than PD patients who adopt postural first strategy to improve neural efficiency, so as to compensate for PD-related automaticity defects and ensure safe walking.

## Data availability statement

The raw data supporting the conclusions of this article will be made available by the authors, without undue reservation.

## Ethics statement

This study was performed in accordance with the Code of Ethics of the World Medical Association (Declaration of Helsinki) for experiments involving humans. The study was approved by the Tianjin Huanhu Hospital Ethics Committee (Approval no. of Ethics Committee: 2019-31). This study was registered in the China Clinical Trial Registry (Register Number: chiCTR1900022655). The patients/participants provided their written informed consent to participate in this study.

## Author contributions

XZ and YW participated in the design of the study, performed the data analysis and wrote the manuscript. NY, JW, and YC recruited the study subjects and reviewed the manuscript. JL and ZS implemented computer code and supported algorithms. ZZ and YY participated in the design of the study and reviewed the manuscript. JW and JH managed and coordinated the planning and execution of research activities, edited the manuscript. All authors contributed to the article and approved the submitted version.

## Funding

This work is funded by the National Natural Science Foundation of China [U1913208 and 61873135], Tianjin Key Medical Discipline (Specialty) Construction Project (No. TJYXZDXK-052B), Tianjin Research Innovation Project for Postgraduate Students [2021YJSS161], and Tianjin Health Research Project [TJWJ2022MS033].

## Conflict of interest

The authors declare that the research was conducted in the absence of any commercial or financial relationships that could be construed as a potential conflict of interest.

## Publisher's note

All claims expressed in this article are solely those of the authors and do not necessarily represent those of their affiliated organizations, or those of the publisher, the editors and the reviewers. Any product that may be evaluated in this article, or claim that may be made by its manufacturer, is not guaranteed or endorsed by the publisher.
